# Self-Inflicted Needle Injuries to the Eye: A Curing Pain

**DOI:** 10.1155/2015/960579

**Published:** 2015-02-24

**Authors:** Shahrokh Amiri, Asghar Arfaei, Sara Farhang

**Affiliations:** ^1^Research Centre of Psychiatry and Behavioral Sciences, Tabriz University of Medical Sciences, Tabriz, Iran; ^2^Razi Hospital, Elgoli Road, P.O. Box 5456, Tabriz, East Azerbaijan, Iran

## Abstract

There are few reports of severe self-injury to eyes in patients with schizophrenia. We report on a 41-year-old woman, primarily visiting for symptoms of endophthalmitis resulting from self-inflicted needles. Further evaluations established the diagnosis of schizophrenia because of arguing and commenting on auditory hallucinations and negative symptoms including social isolation, decreased self-care, blunt affect, and a monotone voice. The patient had been suffering from auditory hallucinations for several years and found relief in bodily pain caused by needles. The patient received 6 mg of risperidone. Hallucinations were resolved and self-injury behaviour was not repeated.

## 1. Introduction

Deliberate injuries to major organs are rare, typically observed in patients with serious mental illness and often resulting in permanent loss of an organ or its function [1]. The majority of patients with serious self-inflicted injuries to the eye were diagnosed as suffering from schizophrenia spectrum psychosis [2–10]. This could happen early in the course of the disorder as most of the injuries that caused loss of vision happened in the first episode of psychosis [11]. The most vulnerable patients are those experiencing command hallucinations, having religious preoccupations, and using illicit substances and those who are socially isolated [12, 13].

There are about 30 reports of self-inflicted injuries to eyes from different parts of the world. Here we report on the case of an Iranian woman with schizophrenia and self-inflicted needle injury to the eye.

## 2. Case Report

A psychiatry consult was required for a 41-year-old woman from the Tertiary Ophthalmology Hospital, Tabriz University of Medical Sciences. She was admitted with symptoms of endophthalmitis in the right eye and further evaluations reported the presence of multiple sewing needles in head and neck. Vision of both eyes was intact and no abnormal sign was observed in neurological examinations. After appropriate treatments for endophthalmitis, the patient was transferred to Razi Hospital, the university mental hospital, for further evaluations.

The patient was a graduated, married housewife. She had two daughters, who had married and moved out. Her husband was military staff and they have been married for 22 years. The patient was admitted with stable vital signs. She was uncooperative and indifferent about being admitted to a hospital. She had no special complaint except for a mild headache and gave short answer to direct questions about her situation.

The most important findings in her mental status were a decreased eye contact, a monotone voice, no spontaneous speaking, paucity of answers, blunt affect, and no insight. She described herself as depressed and tired but denied any delusion or hallucinations. She also did not answer direct questions about the needles. Her family explained that they had noticed a gradual change in her behaviour during the last year or two, beginning with decreased interest in family conversations which were almost her only social relationships as none of their relatives live in her home town. Later they noticed that she had become less interested in cooking, housework, and her own hygiene. She had also complained about intrusive voices coming from neighbours a few times, but her family did not recognize the reason or the importance of such complaints. Persistence of these problems motivated the family to see a general physician. A diagnosis of depression was made based on her symptoms and the doctor prescribed antidepressant medications to help her with sleep problems. She could not take the pills regularly and her symptoms continued, while her daughter noticed the red eye and took her to the hospital.

Past medical history and family history were unremarkable. The patient's family described her premorbid personality to be more or less shy but eager to communicate with relatives, introverted, and tolerant. No information was available about her developmental history. She got married in a traditional way, in which a premarital relationship is not encouraged. She seldom complained of marital conflict but her relationship with her husband was very limited because of his busy life.

**Figure 1:**
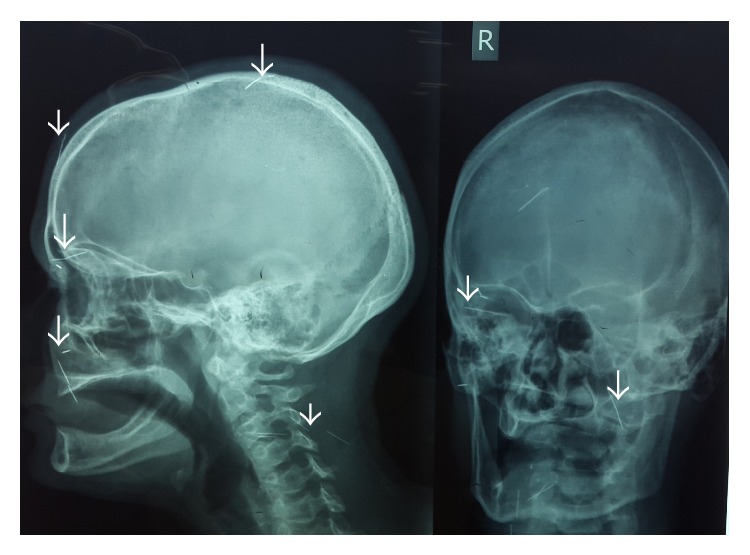
Skull X-ray showing at least eleven needles (some are arrowed).

X-ray anteroposterior and lateral views showed the presence of multiple sewing needles in the head and neck (Figure 1). Besides the described findings in her eye, several indurations resembling an old self-infliction were palpable in her neck, while the skin was intact.

Based on the described information, a preliminary diagnosis of psychotic disorder not otherwise specified was made. Risperidone was initiated with 1 mg and increased to 6 mg within two weeks, along with daily visits. Following correction of her sleep pattern, the patient started to cooperate. She could explain that she could hear the voices of neighbours arguing about her and sometimes about her daughters. She did not plan to do something in return as she was sure it would not work; the neighbours talked about her just after her marriage when she moved to the military facility with her husband. They questioned her loyalty to her husband but stopped shortly after she gave birth to her first daughter. She could not talk about these issues with her husband because he had already warned her not to get involved with neighbours.

She began hearing these voices after the marriage of her youngest daughter, when she felt lonesome a few days after the ceremony. This time, she could hear their voices clearly, even in the hospital, but they were more frequent at home where she was alone most of the day. The humiliating voices never commanded her but made her very upset. She did not feel like doing anything as she felt a lot of pressure. She had no explanations for not taking the prescribed medications, and it was considered as a manifestation of avolition. The only way to get rid of this pain was to inflict pain on her body using needles. This could stop voices for days. She did not offer any special reasons for inflicting damage to her eye using needles.

The diagnosis of schizophrenia undifferentiated was established. The treatment regimen included 6 mg risperidone daily and 2 mg biperiden because of neuroleptic-induced Parkinsonism, which resolved within two days. Supportive psychotherapy was provided to the patient during which she mostly felt like talking about her past. During these sessions, the patient was allowed to talk about her concerns and was encouraged and assisted with every-day life problems and the improvement of her quality of life. Admission continued for two more weeks and her behaviour was closely monitored. She reached a satisfying level of self-care and no self-mutilation attempts were observed. After psychoeducation for the family, the patient was discharged. The psychoeducation included information about the disorder, treatment, and relapse prevention. She could not attain the planned psychotherapy sessions but was taking medications regularly in a one-month follow-up and no positive symptoms of schizophrenia were reported.

## 3. Discussion

Reported cases of self-inflicted injuries to eyes are mainly restricted to psychotic patients [14–17]. All of the reported cases were visited by an ophthalmologist for the first time. The presented patient here, owing to her daughter, received timely ophthalmologic intervention and did not lose her vision. However, family members did not care about her mental symptoms in the same way and psychiatric intervention began late. The first episode of psychosis was also missed, mostly because of low emotional involvement of her husband. Social isolation determined by him was a precipitating and perpetuating factor. Their lack of intimacy was very obvious. This even continued during admission and he hardly ever visited the patient. This emotional atmosphere not only seems to play a significant role as a predisposing factor but also could perpetuate the disorder because emotional isolation and low levels of support during stressful events may worsen the prognoses.

Psychodynamic formulations have been proposed for this condition, such as castration fears, failure to resolve oedipal conflicts, repressed homosexual impulses, severe guilt, and self-punishment [17, 18]. The latter two seem to be appropriate for the case reported here. Additionally, the patient suffered too much from her psychotic symptoms and found relief in bodily pain. Similar to many other patients, stigma toward mental illness in Iran seems to be the most important reason for the delayed diagnosis [19, 20]. The situation may be more complicated, as in Muslim communities mental illness may be perceived as a punishment from God [21]. Also in Islamic rules fidelity to the family and husband are emphasized, which are among the core beliefs of Iranian woman. Therefore, experiencing psychotic symptoms with the theme of infidelity imposes a very high mental load.

Most of the reported cases of self-inflicted mutilation of the eyes concern men who often have criminal records and histories of several forms of self-harm [14, 22–24]. This reported case shows some differences from previous reports but the most important difference was the patient's fortunate and timely diagnosis of injury to her eyes. Regardless of severity and number of injuries to eyes, self-injury because of psychosis should be among differential diagnosis of such patients, as timely treatment can improve their outcome.
